# Supplementary material for: Algerian Workers’ Exposure to Mycotoxins—A Biomonitoring Study
Source: Int J Environ Res Public Health. 2023 Aug 12;20(16):6566. doi: 10.3390/ijerph20166566 (PMC10454754; doi:10.3390/ijerph20166566)
Supplement: Supplementary file 1 [file ijerph-20-06566-s001.zip › ijerph-2468823-supplementary.pdf]

Algerian Workers’ Exposure to Mycotoxins—A Biomonitoring Study

Marta I. Mendes [1] , Sara C. Cunha [1,*] , Iméne Rebai [2] and José O. Fernandes [1,*]

1 LAQV-REQUIMTE, Laboratory of Bromatology and Hydrology, Department of Chemical Sciences, Faculty of Pharmacy, University of Porto, 4050-313 Porto, Portugal; up201809668@ff.up.pt

2 Laboratory of Toxicology, Faculty of Medicine, Salah Boubnider University 3, Constantine 5000, Algeria; imene.rebai@univ-constantine3.dz

* Correspondence: sara.cunha@ff.up.pt (S.C.C.); josefer@ff.up.pt (J.O.F.);

Tel.: +351-220428639 (S.C.C.)

Table S1- Sample characterization

SAMPLE Nº	AGE	BMI	HABITATION	SAMPLE Nº	AGE	BMI	HABITATION	SAMPLE Nº	AGE	BMI	HABITATION
1	40	22.64	RURAL	1	53	24.07	URBAN	65	33	25.80	URBAN
2	58	24.57	RURAL	2	41	27.16	URBAN	66	35	16.69	URBAN
3	43	22.22	URBAN	3	48	26.83	URBAN	67	38	22.49	URBAN
4	38	26.85	URBAN	4	39	28.09	URBAN	68	47	24.66	URBAN
5	49	22.09	URBAN	5	52	23.74	URBAN	69	39	29.32	URBAN
6	52	25.69	RURAL	6	35	26.57	URBAN	70	29	26.78	URBAN
7	44	25.76	URBAN	7	29	23.46	URBAN	71	36	18.76	URBAN
8	57	30.46	URBAN	8	32	26.03	URBAN	72	58	23.88	URBAN
9	44	26.85	URBAN	9	32	26.12	URBAN	73	36	16.01	URBAN
10	49	26.23	RURAL	10	48	29.75	URBAN	74	28	24.80	URBAN
11	39	24.59	URBAN	11	43	23.96	URBAN	75	33	26.53	RURAL
12	49	24.07	URBAN	12	50	23.96	RURAL	76	48	27.34	URBAN
13	52	24.22	RURAL	13	32	26.03	URBAN	77	52	25.88	URBAN
14	35	21.63	URBAN	14	39	23.31	URBAN	78	36	24.64	RURAL
15	47	21.30	URBAN	15	57	30.46	URBAN	79	29	21.88	URBAN
16	45	28.62	URBAN	16	34	24.30	-	80	45	25.00	URBAN
17	58	25.54	URBAN	17	42	27.16	URBAN	81	35	26.87	URBAN
18	39	23.18	URBAN	18	35	23.89	URBAN	82	49	30.72	URBAN
19	50	21.45	RURAL	19	47	20.28	URBAN	83	59	21.22	URBAN
20	37	22.21	URBAN	20	46	27.74	URBAN	84	35	22.20	URBAN
21	39	30.30	URBAN	21	39	24.82	URBAN	85	41	24.77	URBAN
22	44	24.54	URBAN	22	45	26.83	RURAL	86	45	25.53	URBAN
23	47	25.31	URBAN	23	42	21.97	URBAN	87	42	26.12	URBAN
24	44	23.55	URBAN	24	33	34.60	RURAL	88	45	20.90	URBAN
25	46	25.21	RURAL	25	32	22.96	URBAN	89	54	25.14	URBAN
26	34	27.78	URBAN	26	46	27.44	URBAN	90	44	25.93	URBAN
27	51	19.52	URBAN	27	60	22.77	URBAN	91	47	26.30	URBAN
28	41	24.22	RURAL	28	38	20.66	URBAN	92	45	24.61	URBAN
29	60	30.00	URBAN	29	28	20.28	URBAN	93	34	18.59	URBAN
30	36	18.59	URBAN	30	49	25.50	URBAN	94	46	22.22	URBAN
31	42	21.80	URBAN	31	49	24.65	URBAN	95	53	27.59	URBAN
32	34	25.00	URBAN	32	40	26.73	URBAN	96	45	26.12	URBAN

__Table S2__- LC-MS/MS parameters for the mycotoxins analysed

Mycotoxin	Retention time (min)	Precursor ion (Da)	Product ions (Da)	Cone energy (V)	Collision energy (kV)
$^{13}C_{15}$DON	7.30	312.2	198.0	20	27
			265.0	20	27
			292.0	20	20
DON	9.8	297.0	203.3	22	13
			249.0	20	11
OTAd5	7.13	409.0	239.0	32	22
			363.0	32	22
OTA	7.19	404.0	239.1	28	24
			358.1	26	18
FB_1	6.65	722.5	334.2	46	40
			352.4	44	36
AFB_1	6.2	313.0	241.4	47	36
			285.3	47	23
AFB_2	6.14	315.0	259.2	47	29
			287.3	50	27
T-2	6.96	484.0	214.5	21	18
			245.2	23	15
HT-2	6.65	441.9	215.3	16	12
			263.2	16	13
DON-3-G	7.70	493.0	326.0	80	35
			382.0	80	27
			440.0	82	25
DOM-1	8.19	281.0	109.0	15	15
			148.0	15	15
			245.0	15	15
α-ZEL	9.54	321.1	285.0	15	12
			303.0	13	10
			304.0	13	10
ZEN	7.36	321.0	189.0	22	15
			302.7	20	15

$^{13}C_{15}$DON, 13 C15deoxynivalenol; DON, deoxynivalenol; OTAd5, ochratoxin A-(phenyl-d5); OTA, ochratoxin A; FB1, fumonisin B1; AFB_1, aflatoxin B1; AFB_2, aflatoxin B2; T-2, T-2 toxin; HT-2, HT-2 toxin; DON-3-G, deoxynivalenol-3-glucoside; DOM-1, deepoxy-deoxynivalenol; α-ZEL, α -zearalenol; ZEN, zearalenone.

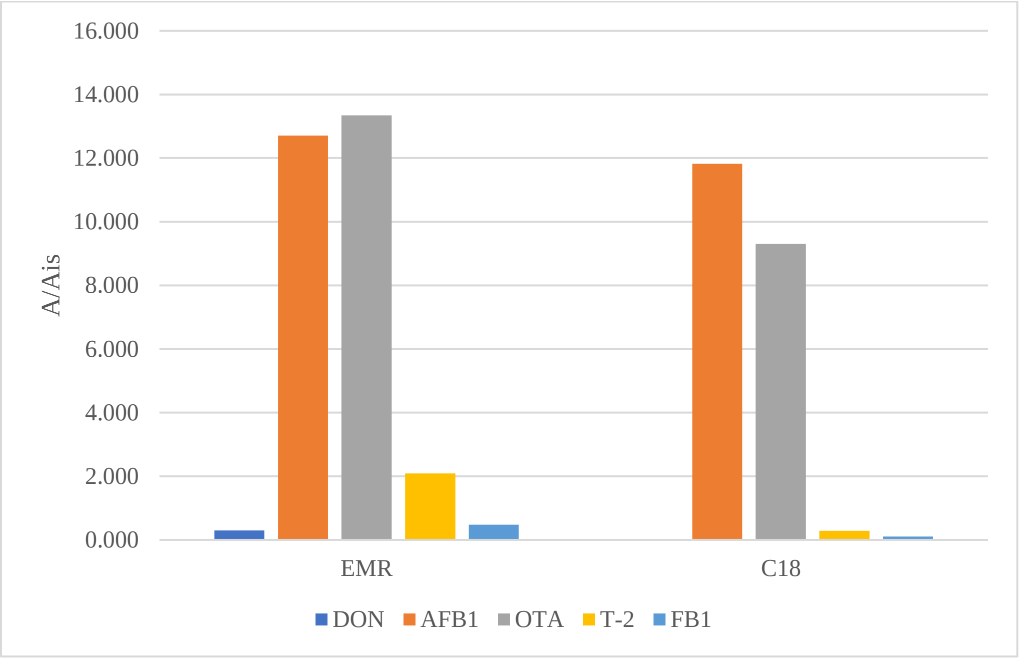

Figure S1- Comparison of sorbents and extraction solvents in QuEChERS extraction

DON, deoxynivalenol; AFB_1, aflatoxin B1; OTA, ochratoxin A; T-2, T-2 toxin; FB1, fumonisin B1
